# In vivo theranostics with near-infrared-emitting carbon dots—highly efficient photothermal therapy based on passive targeting after intravenous administration

**DOI:** 10.1038/s41377-018-0090-1

**Published:** 2018-11-21

**Authors:** Xin Bao, Ye Yuan, Jingqin Chen, Bohan Zhang, Di Li, Ding Zhou, Pengtao Jing, Guiying Xu, Yingli Wang, Kateřina Holá, Dezhen Shen, Changfeng Wu, Liang Song, Chengbo Liu, Radek Zbořil, Songnan Qu

**Affiliations:** 1 State Key Laboratory of Luminescence and Applications, Changchun Institute of Optics Fine Mechanics and Physics Chinese Academy of Sciences, 3888 Dong Nanhu Road, Changchun, 130033 China; 2 School of Physical Sciences, University of Chinese Academy of Sciences, Beijing, 100190 China; 3 Department of Biomedical Engineering, Southern University of Science and Engineering, Shenzhen, Guangdong 518055 China; 4 Research Laboratory for Biomedical Optics and Molecular Imaging, Institute of Biomedical and Health Engineering, Shenzhen Institutes of Advanced Technology, Chinese Academy of Sciences, Shenzhen, 518055 China; 5 Jilin Provincial Tumor Hospital, Changchun, China; 6 Department of Physical Chemistry, Faculty of Science, Regional Centre of Advanced Technologies and Materials, Palacký University Olomouc, 783 71 Olomouc, Czech Republic; 7 CAS Key Laboratory of Health Informatics, Shenzhen Institutes of Advanced Technology, Shenzhen, 518055 China

## Abstract

Carbon dots that exhibit near-infrared fluorescence (NIR CDs) are considered emerging nanomaterials for advanced biomedical applications with low toxicity and superior photostability and targeting compared to currently used photoluminescence agents. Despite progress in the synthesis of NIR CDs, there remains a key obstacle to using them as an in vivo theranostic agent. This work demonstrates that the newly developed sulfur and nitrogen codoped NIR CDs are highly efficient in photothermal therapy (PTT) in mouse models (conversion efficiency of 59%) and can be readily visualized by photoluminescence and photoacoustic imaging. The real theranostic potential of NIR CDs is enhanced by their unique biodistribution and targeting. Contrary to all other nanomaterials that have been tested in biomedicine, they are excreted through the body’s renal filtration system. Moreover, after intravenous injection, NIR CDs are accumulated in tumor tissue via passive targeting, without any active species such as antibodies. Due to their accumulation in tumor tissue without the need for intratumor injection, high photothermal conversion, excellent optical and photoacoustic imaging performance, and renal excretion, the developed CDs are suitable for transfer to clinical biomedical practice.

## Carbon dots: Lighting up for cancer therapy and diagnosis

‘Carbon dot’ nanoparticles composed largely of carbon, but doped with sulfur and nitrogen, accumulate within cancer cells and absorb near-infrared laser light to generate cell-killing heat. The procedure was developed and tested by researchers in China and the Czech Republic, led by Songnan Qu at the Changchung Institute of Optics, Fine Mechanics and Physics. The nanoparticles accumulated passively in tumors after intravenous injection into mice, with no specific targeting methods required. Tumors were effectively eradicated by the highly efficient conversion of the light energy into heat. The nanoparticles are biocompatible and are eventually excreted harmlessly in urine. In addition to the potential for treating cancer, diagnosis can also be assisted using the particles’ fluorescent properties. This combination of therapy and diagnosis – ‘theranostics’ – can now be further developed and explored for use in patients.

## Introduction

Cancer is one of the most severe threats to human health. Cancer diagnosis and treatment are issues of acute focus due to high-cancer mortality. Traditional treatment methods (including radiotherapy, chemotherapy, and surgery) have significant side effects, such as physical disorders, reduced immunity, organ damage, and damage of normal tissue. In addition, the high costs of anticancer drugs and cancer treatments overwhelm many cancer patients. In addition to pain from the illness, many cancer patients also suffer from substantial mental distress due to the financial burden. Therefore, it is urgent and of significant value to develop effective cancer treatment methods and drugs that have few side effects and are low-cost and easy to synthesize on a large scale.

Fluorescence (FL) imaging, photoacoustic (PA) imaging, and photothermal therapy (PTT) have attracted increasing interest because they are noninvasive and nonionizing and cause little tissue damage^1–5^. These techniques require agents that have low toxicity, high absorption coefficients, strong fluorescence, and photothermal conversions within the biological transparency window (650–950 nm)^6,7^, and the ability to accumulate at the tumor site^8^. The FL imaging, PA imaging, and PTT agents that are found in the literature are typically based on organic dyes, noble metal nanoparticles, and semiconductor oxides^9–25^. Organic dyes have weak thermostability and photostability, while excretion of noble metal nanoparticles and semiconductor oxides from the body via the renal system is typically difficult, thereby exposing the patient to a risk of visceral deposits and the potential toxicity of heavy metal elements^26,27^. The United States (US) Food and Drug Administration (FDA), to date, has not approved any inorganic nanoparticles for clinical PTT or PA applications.

Due to the abundance, biocompatibility, and nontoxicity of carbon, carbon nanomaterials such as carbon nanotubes and graphene are of keen interest for PTT and PA applications^28–30^. Yang et al. reported on a biological application of graphene, namely, the first successful use of carbon nanomaterials for efficient in vivo PTT via intravenous administration (2 W cm^−^^2^)^28^. Moon et al. used carbon nanotubes for PTT in mice under 808 nm irradiation (3.8 W cm^−2^)^30^. The ideal PTT and PA imaging agents for in vivo applications require high absorption coefficients that are within the biological transparency window (650–950 nm) and rapid excretion from the body via renal filtration. However, these reported carbon nanomaterials exhibited relatively low-absorption coefficients in the red to near-infrared (NIR) region, which substantially restricted their PA imaging and PTT performance. Furthermore, the sizes of carbon nanotubes and graphene exceed the renal threshold in all their dimensions^31^. Carbon dots (CDs), which are emerging luminescent carbon nanomaterials with sizes of less than 10 nm, are considered zero-dimensional carbon-based nanomaterials. CDs have the following distinct advantages: low-cost, low-toxicity, low-environmental impact, strong fluorescence, and high thermostability and photostability^32–49^, which make them good candidates for in vivo and in vitro biological applications. Moreover, CDs have highly suitable biodistribution profiles in mice^32^. The main absorption bands of CDs, to date, are typically in the ultraviolet (UV)-to-green region of the spectrum. Tuning these bands to the red-to-NIR region to obtain acceptable performance for FL imaging, PA imaging, and PTT remains challenging^50–59^. Lan et al. reported CDs with a maximum absorption band at 526 nm and a photothermal conversion efficiency of up to 58.2% under a 635-nm laser (2 W cm^−2^)^58^. Zheng et al. synthesized NIR-emitting CDs with maximum absorption at 370 nm and a photothermal conversion efficiency of 38.7% under an 808-nm laser (2 W cm^−2^)^59^.

However, the absorption coefficients of these reported CDs were significantly reduced in the red-to-NIR region. Thus, all published studies of CD-based PTT were performed under relatively high irradiating power densities (≥2 W cm^−^^2^), which increased the risk of tissue damage^8^. Moreover, these CD-based PTT events were initiated via intratumor injection, which broke the outer tissue of the tumor and posed the risk of cancer cells spreading to other parts of the body. To the best of our knowledge, red-emitting CDs of only one type could accumulate at the tumor site after intravenous injection for PA imaging; this result was reported by Ge. These CDs had their maximum absorption at 470 nm and a photothermal conversion efficiency of 38% under 671 nm laser (2 W cm^−2^)^56^. However, the PTT performance of these CDs required intratumor injection. Thus, for clinical FL imaging, PA imaging, and PTT application, the development of CDs with intense absorption in the red to NIR region and strong NIR fluorescence, high photothermal conversion efficiency, and the ability to accumulate within the tumor after intravenous injection is of significant value.

Our group is engaged in a long-term endeavor to develop CDs that have a controllable optical bandgap from citric acid and urea. In this work, we prepared novel NIR-emitting CDs from citric acid and urea using the solvothermal method with dimethylsulfoxide (DMSO) as both the solvent and the sulfur-doping source. As prepared, the CDs show a broad and strong absorption band in the red to NIR region with a maximum absorption coefficient at 600 nm and a mass absorption coefficient in the red to NIR region that is much higher than that of graphene oxide. Strong NIR emission that peaked at 720 nm and high photothermal conversion efficiency (59.19%) were simultaneously achieved under 655-nm laser irradiation.

The CDs, as prepared, showed excellent biocompatibility and low toxicity, and were quickly cleared through the renal excretion system in mice after intravenous injection. More importantly, the CDs accumulated at the tumor site in vivo after intravenous injection and could be visualized by NIR FL imaging and PA imaging. Based on these properties, we achieved acceptable PTT performance for tumors in mice via intravenous injection of the CDs under 655-nm laser irradiation at 1W cm^−2^. These attractive properties demonstrate that the CDs, as prepared for this work, could be suitable agents for FL imaging, PA imaging, and PTT for cancer diagnosis and treatment, and are promising agents for CD-based clinical applications.

## Materials and methods

### Synthesis of CDs

First, 2 g citric acid and 6 g urea were dissolved in 30 mL DMSO, heated at the temperature of 160 °C for 4 h under solvothermal conditions in a reaction autoclave, and cooled to room temperature. We acquired dark solution and mixed with twice its volume of ethanol solution. Then, it was centrifuged at 8000 r min^−1^ for 5 min to remove residual solvents and eventual organic molecular byproducts. Finally, the precipitate was collected, freeze-dried into a dark product, and ground into a powder.

Materials: Citric acid and urea were purchased from Aladdin, and DMSO was purchased from Guangfu.

### Characterizations

Transmission electron microscopy (TEM) was performed on an FEI Tecnai-G2-F20 transmission electron microscope (200 kV). A Multimode 8 (Bruker Co.) instrument was used for collect the AFM imaging. An Inca X-Max instrument was used for perform the EDS and elemental mapping. The XPS analyses were conducted on an ESCALAB MK II X-ray photoelectron spectrometer using Mg as the exciting source. Fourier transform infrared (FT-IR) spectra (Figure S8) were obtained with a Perkin–Elmer spectrometer. A Shimadzu UV-3101PC spectrophotometer was used for collected the UV–visible absorption spectra, and the emission spectra were acquired from a Horiba Jobin Yvon Fluorolog-3 spectrometer (Xenon lamp excitation). The fluorescence imaging of cell was collected from an Olympus FV1000 confocal laser scanning microscope. The laser (655 nm) was generated from cnilaser MD-655NM-HS-2W-16060512. Photothermal images were captured by a FLIR E50 (FLIR Systems, Inc.) thermal imaging camera.

### Photothermal effect measurements

UNT-T323 digital thermometers with a K-type thermocouple was used for measured the photothermal effect data. A 0.5 mL volume of CDs aqueous solution was introduced into a quartz cuvette, then the cuvetee was irradiated with the laser (655 nm) for 10 min, the power density is 1 W cm^−2^. Pure water was used as a negative control. The thermocouple probe linked the digital thermometers inserted into the CDs aqueous solution, which was perpendicular to the light path. So the temperature of CDs aqueous solution could be recorded at 10-s intervals by the digital thermometer, which were further investigated. The temperature change of CDs aqueous solution (200 μg mL^−1^, 0.5 mL) was regarded as the function of time under 655 nm lasers irradiation, until it reached the room temperature. Photothermal conversion efficiency reached approximately 59.2% according to the data that were obtained (Figure S4).

### Potential biotoxicity, fluorescence, and PA imaging of CDs

The environment contains 5% CO_2_ (the temperature is 37 °C), and HeLa cells, 4T1 cells, and HepG2 cells were cultured in Dulbecco’s modified Eagle medium with 10% fetal bovine serum and 1% penicillin/streptomycin. And the 3-[4,5-dimethylthiazol-2-yl]-2,5-diphenyltetrazolium bromide (MTT) assays were used for in vitro cell activities evaluation. These cells were seeded into U-bottom 96-well cell culture plates. The density of cells was 5 × 10^4^ well^−1^.

Then, the media were removed and these cells in wells were incubated with CDs aqueous solution at various concentrations (0–1 mg mL^−1^) at 37 °C for 24 h. After 24 h the MTT (20 μL, 5 mg mL^−1^) was added and cells were incubated in the each well for 4 h. Absorbance (OD570 nm) of each well was measured by microplate reader and the cell viability was calculated via the following equation ($\overline {A_t}$ is the mean absorbance value of the treatment group and $\overline {A_c}$ is the mean absorbance value of the control group):

1 $${\mathrm{Cell}}\,{\rm{viability}}\,\left( {\mathrm{\% }} \right) = \frac{{\overline {A_{\rm{t}}} }}{{\overline {A_{\rm{c}}} }} \times 100\%$$

For cell viability detection after exposure to the laser, the cells were costained with a live/dead cell double staining kit to monitor viable and dead cells with the confocal fluorescence microscope. The double staining kit contained acetoxymethyl ester of calcein which stained only viable cells with green fluorescence, and propidium iodide which stained only dead cells with red fluorescence.

All animal experiments were conducted according to the animal research guidelines provided by the Animal Care and Use Committee at the University of Macau. ICR mice used in all experiments were purchased from Beijing HFK Bioscience Co. Ltd. For CD metabolism and tumor targeting in vivo, the mice received an intravenous injection of 0.1 mL (2000 μg mL^−1^) aqueous CD solution.

FL images of mouse major organs and urine were acquired using a Fluor Vivo Model-300 in vivo optical imaging system. The excitation wavelength was 639–713 nm, and the fluorescence collection channels were 714–780 nm. Urine was collected using a mouse urine collector. Tumor fluorescence images were collected using an ORCA-Flash4.0 V2 Digital CMOS camera on the whole body of the mouse. The excitation laser (655 nm) was generated from cnilaser MD-655NM-HS-2W-16060512 (10.6 mW cm^−2^) and emitted light was further filtered through a 700-nm longpass filter that was coupled to the CMOS camera. The exposure time is 400 ms, and the images were further processed with the ImageJ image analysis software.

For PA imaging, tumor-bearing mice with i.v. injected CDs aqueous solution were kept under anesthesia. Tumor area were PA imaged by using acoustic-resolution PA microscopy system^21^ at 1, 2, 3, 4, and 24 h post i.v. injection of CDs aqueous solution.

### In vivo PTT

The H22 tumor models of the ICR mice were generated by subcutaneous injection of H22 hepatoma ascites in 100 μL into the dorsal area of each female ICR mouse. These mice were randomized into three groups (*n* = 10 per group) when the volume of these tumor xenografts reached 150–250 mm^3^. After inhaled of 2% isoflurane, the mice were anesthetized. The PTT treatment group was labeled (i) and the control groups (ii) and (iii). Mice belong to group (i) were intravenously injected with CDs aqueous solution (0.2 mL, 1000 μg mL^−1^), and irradiated under NIR laser (the laser wavelength is 655 nm, power density is 1 W cm^−2^, 5 min) after 3 h. The mice belong to group (ii) were intravenously injected with phosphate-buffered saline (PBS) (0.2 mL) and irradiated the NIR laser which was same as the group (i). The mice belong to group (iii) were intravenously injected with CDs aqueous solution (0.2 mL, 1000 μg mL^−1^) with no laser irradiation. A FLIR E50 (FLIR Systems, Inc.) thermal imaging camera was used for recorded the temperature changes of tumor sites. Sizes of tumors were measured by using a digital caliper, and volumes were calculated via the following equation:

2 $${\mathrm{Volume}} = 1/2 \times {\mathrm{Length}} \times {\mathrm{Width}}^2$$

### Histopathological evaluation

For histological analysis, the organs (heart, liver, spleen, lung, and kidney) were fixed in 10% formalin, then embedded in paraffin. Slices of these organs from the mice were stained with hematoxylin and eosin (H&E). The histological sections were imaged by an optical microscope.

## Results and discussion

**Fig. 1:**
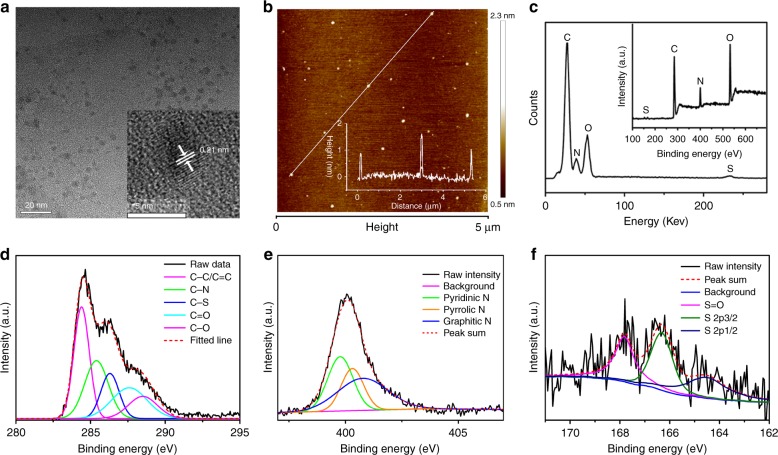
Morphology, composition, and structural characterization of the CDs. **a** A TEM image of the CDs. The inset shows an HRTEM image of the corresponding CDs. **b** An AFM image. **c** EDS and XPS survey spectra of the CDs. Deconvolutions of high-resolution **d** C 1 s, **e** N 1 s, and **f** S 2p XPS spectra of the CDs

The morphology and structure of the CDs were investigated by TEM and atomic force microscopy (AFM). As shown in the TEM images (Fig. 1a), the diameters of the CDs range from 2 to 5 nm. The high-resolution TEM images (inset of Fig. 1a) show the circular shape of the CDs with clearly visible lattice fringes that are 0.21 nm wide, which correspond to the (100 nm) plane of graphene^60^. The heights of the CDs ranged from 0.5 to 2 nm according to AFM observation (Fig. 1b). Thus, the CDs are shorter than they are wide.

The chemical compositions of the CDs were investigated by energy dispersive X-ray spectrometry (EDS) and X-ray photoelectron spectroscopy (XPS), which revealed the presence of C, N, S, and O elements (Fig. 1c). The atomic ratios of C, N, O, and S in EDS are 45.5%, 31.7%, 21.5%, and 1.2%, respectively. The high-resolution C1s XPS spectra (Fig. 1d) revealed peaks at 284.5, 285.3, 286.2, 287.5, and 288.8 eV, which were assigned to the C–C or C = C, C–N, C–S, C = O and C(O)–O bonds, respectively^61^. The two XPS peaks that fit O1s at 531.6 and 533.1 eV were attributed to the C = O and C-OH/C-O-C groups (Supplementary Fig. 1). The XPS N1s spectrum fit three peaks at 399.6, 400.3, and 401.0 eV, which were attributed to pyridinic N, pyrrolic N, and graphitic N, respectively (Fig. 1e). The high-resolution S 2p spectra (Fig. 1f) revealed peaks at 164.6, 166.3, and 167.8 eV, which correspond to S 2p1/2, S 2p3/2, and the S = O bond and indicate the incorporation of sulfur into the CDs. These results demonstrate the S, N-doped property of the CDs. The zeta potential of the CDs in the aqueous solution was −20.1 mV, owing to the presence of abundant functional groups with negative charge. The surface functional groups of the CDs are detected via FT-IR (Supplementary Fig. 2). Absorption bands at 3100–3300 cm^−1^ are assigned to *v* (O–H), at 1550–1770 cm^−1^ to *v* (C = N) and *v* (C = O), and at 1000–1030 cm^−1^ to *v* (C–S). Thus, the predominant surface functional groups are carboxyls and carbonyls, which is in accordance with the XPS data and negative zeta-potential that was measured.

**Fig. 2:**
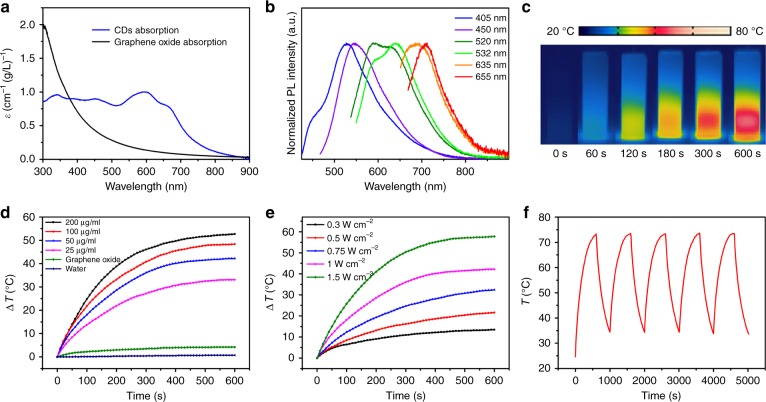
Photophysical and photothermal properties of the CDs. **a** Absorption spectra of the CDs and a graphene oxide aqueous solution with the same mass concentration (200 μg mL^−1^). **b** Emission spectra of CDs that are excited at various wavelengths in dilute aqueous solution that were obtained using a Horiba Jobin Yvon Fluorolog-3 spectrometer with Xenon lamp excitation. **c** Photothermal images of CD aqueous solutions (200 μg mL^−1^) that were captured at various times under 655-nm laser irradiation at a power density of 1W cm^−2^. **d** Temperature evolutions of CD aqueous solutions of various concentrations, graphene oxide aqueous solution at 50 μg mL^−1^, and pure water under 655-nm laser irradiation at a power density of 1 W cm^−2^. **e** Temperature evolutions of CD aqueous solutions (50 μg mL^−1^) at various power densities. **f** Temperature curves of aqueous CD aqueous solution (200 μg mL^−1^) under five cycles of photothermal heating under 655-nm laser irradiation (1 W cm^−2^)

In the UV–vis spectrum, a broad absorption of the CDs aqueous solution was extended from the UV to the NIR region with absorption peaks at 340, 455, 605, and 650 nm. The mass absorption coefficient of the CDs in the red to NIR region is much higher than that of graphene oxide (Fig. 2a). The aqueous CD solution also exhibited excitation-dependent fluorescence from blue to NIR emissions under excitation from UV to red light (Fig. 2b). A strong NIR emission that was centered at 720 nm and had a PLQY (which refers to the number of emitted photons divided by the number of absorbed photons) of 0.2% was observed under a 655-nm excitation in dilute aqueous solution (Supplementary Fig. 3). The fluorescence intensity at 720 nm and the zeta potential of the CDs aqueous solution did not change significantly from pH 5 to pH 9 (Supplementary Fig. 4), thereby demonstrating that the CDs can play the role of NIR-fluorescent probes for bioimaging in vivo.

In our previous work, we synthesized efficient orange-emitting CDs with a main absorption band that covered the green to yellow region^52^. The synthesis methods of the orange-emitting CDs and the CDs that were prepared in this work were very similar. The only difference between the two synthetic routes was the solvothermal conditions of the solvents. DMSO and dimethylformamide (DMF) are both polar aprotic solvents; however, DMSO contains sulfur. Based on the EDS and XPS results, DMSO is both a sulfur-doping source and a solvent. In previous publications, S-doping lowered optical bandgaps^5,51^. CD absorption bands with longer wavelengths were prepared in DMSO instead of in DMF; from this, we infer that S-doping introduces a lower energy level, thereby reducing the optical bandgap and contributing to the strong absorption band in the red to NIR region and to NIR emissions under 655-nm excitation.

The photothermal performance of the CDs in aqueous solution (0–200 μg mL^−1^) was examined using a 655-nm laser at 1 W cm^−2^. The photothermally induced temperature enhancement of the aqueous CD solution (200 μg mL^−1^) was visualized using an infrared thermal mapping apparatus (Fig. 2c). Temperature of the aqueous CD solutions at 25, 50, 100, and 200 μg mL^−1^ quickly increased by 33.1, 42.2, 48.4, and 52.7 °C from room temperature after irradiated for 600 s, respectively (Fig. 2d). In contrast, the temperatures of pure water and the commercially available graphene oxide aqueous solution (50 μg mL^−1^) increased by less than 4.2 °C under the same conditions.

Figure 2e shows the temperature increases of the 50 μg mL^−1^ aqueous CD solution at various power densities. The temperature of the 50 μg mL^−1^ aqueous CD solution increased by 13.4, 21.7, 32.5, 42.2, and 57.9 °C from room temperature under 655-nm laser irradiation for 600 s at 0.3, 0.5, 0.75, 1.0, and 1.5 W cm^−2^, respectively.

Remarkably, the photothermal conversion efficiency reached approximately 59.2% (CDs: 200 μg mL^−1^, laser: 655 nm, power density: 1 W cm^−2^; Supplementary Fig. 5)^8,9^, which falls among the highest levels for the carbon-based nanomaterials and other inorganic nanoparticles that have been investigated. This photothermal conversion efficacy is comparable to those of other organic nanoparticles^62–64^. Furthermore, the CDs exhibited satisfactory photostability and thermostability under 655-nm laser irradiation. No substantial deterioration of the photothermal performance of the aqueous CD solution (200 μg mL^−1^) was observed after five cycles of irradiation (Fig. 2f). Excellent photothermal performance of the CDs indicates that they are an efficient photothermal agent for PA imaging and PTT applications^52^.

**Fig. 3:**
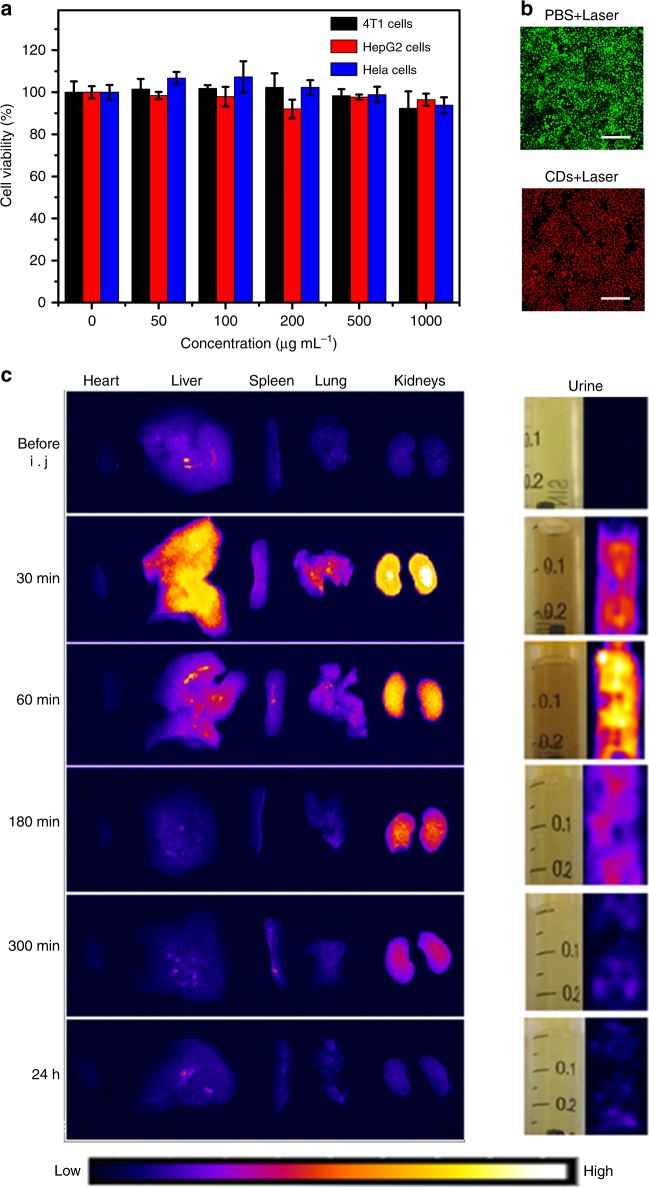
Cytotoxicity and biodistribution of the CDs. **a** Relative cell viabilities of HeLa cells that were incubated with aqueous CD solution at various concentrations (0–1000 μg mL^−1^) for 24 h. **b** Confocal fluorescence images of HeLa cells that were incubated in PBS solution and aqueous CD solution (200 μg mL^−1^) after irradiation by the 655-nm laser at 1W cm^−2^ for 10 min; scale bar: 200 μm. **c** NIR fluorescence images of dissected major organs from mice without and with intravenous injection of aqueous CD solution (0.2 mL, 1000 μg mL^−1^) after various time points (left) and bright field and NIR fluorescence images of urine that was collected at the corresponding time points (right). The NIR fluorescence images were acquired using a Fluor Vivo Model-300 in vivo optical imaging system with xenon lamp excitation. The excitation wavelength was 639–713 nm and the fluorescence collection channels were 714–780 nm

The cytotoxicity of the CDs was examined in HeLa cells, 4T1 cells, and HepG2 cells. According to Fig. 3a, the CDs did not inhibit cell viability at concentrations up to 1000 μg mL^−1^; thus, they are of very low cytotoxicity. In vitro photothermal ablation of cancer cells using the aqueous CD solution was also investigated (Fig. 3b). After 655-nm laser irradiation at 1 Wcm^−2^ for 10 min, all the cells survived in PBS solution; but when in the CDs aqueous solution (200 μg mL^−1^) the cell viability decreased significantly under the same laser irradiation, thereby demonstrating the efficacy of CD-based photothermal ablation of cancer cells in vitro.

Based on the NIR emission properties and in vitro cytotoxicity observations of the CDs, we further examined the biocompatibility and biodistribution of the CDs using NIR FL imaging in vivo. Twenty mice were intravenously injected with aqueous CD solution (100 μL, 2 mg mL^−1^). Major organs (heart, liver, spleen, lung, and kidneys) were excised and studied before and at several time points after (30 min, 1 h, 3 h, 5 h, and 24 h) intravenous injection of the CDs for ex vivo NIR FL imaging to quantify the fluorescence intensity.

The major organs that were not injected with CDs did not show NIR fluorescence under xenon lamp excitation in the 639–713 nm wavelength range. However, the liver and kidneys exhibited strong NIR-fluorescent signals under the same excitation conditions after intravenous injection of the CDs (Fig. 3c). The NIR-fluorescent signal that was acquired from the kidneys at 30 min postinjection was much stronger than those from the other organs; this signal gradually decreased until disappearing after 24 h postinjection, thereby indicating that the CDs mainly accumulated in the kidneys in the first several hours postinjection.

Urine was also collected from mice for NIR FL imaging before and after intravenous injection of the CDs at the corresponding time points. No signal of NIR fluorescence was observed in urine from mice that had not been injected with CDs. However, a strong NIR-fluorescent signal was observed in urine that was collected at 30 min and 1 h postinjection of CDs, which gradually decreased until disappearing after 24 h postinjection. This observation aligns with what we observed in the kidneys. Thus, we conclude that the CDs were quickly excreted through the kidneys after intravenous injection, thereby demonstrating excellent biocompatibility and low or no biotoxicity.

**Fig. 4:**
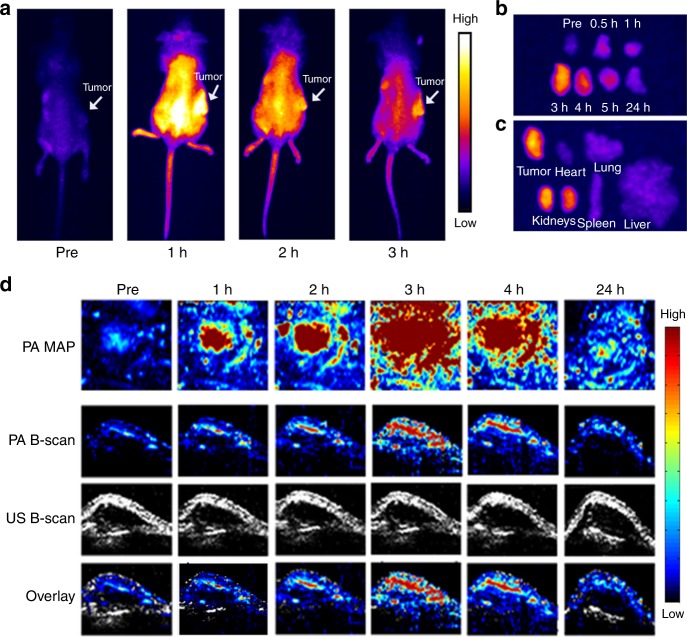
Passive targeting of CDs in vivo. **a** NIR fluorescence images of mouse bodies after intravenous injection of CDs (0.2 mL, 1000 μg mL^−1^) at various time points. **b** NIR fluorescence of H22 tumors that were dissected from mice at various postinjection time points. **c** NIR fluorescence of major organs and H22 tumors that were dissected from mice at 3 h postinjection. NIR fluorescence images of mouse bodies, major organs, and tumors that were acquired using an ORCA-Flash 4.0 V2 Digital CMOS camera, with excitation by 655-nm laser (10.6 mW cm^−2^) that was generated from MD-655NM-HS-2W-16060512. The emitted light was filtered through a 700-nm longpass filter that was coupled with a CMOS camera for NIR imaging. **d** PA MAP images and B-scan PA images of 4T1 tumors in mice after intravenous injection with CDs at various time points

We further examined the in vivo biodistribution of the CDs via NIR FL imaging of mice with and without tumors to evaluate the feasibility of using the CDs for tumor diagnosis and treatment. After intravenous injection of CDs into mice with H22 tumors, the whole body of each mouse gradually exhibited strong NIR fluorescence. At 3 h postinjection, the whole-body NIR fluorescence intensity had decreased substantially, while an NIR-fluorescent signal from the tumor area contrasted strongly with other tissues (Fig. 4a).

Changes to the NIR-fluorescent signal from the tumor tissues were carefully investigated. The tumors were excised at various postinjection time points from live (anesthetized) mice that had or had not received intravenous injection of CDs (Supplementary Fig. 6). The NIR-fluorescent signal from the tumors gradually increased for 2–3 h postinjection. The strongest NIR-fluorescent signal was observed at 3 h postinjection, after which it decreased gradually (Fig. 4b); thus, the maximum accumulation of the CDs in the tumor area occurs at 3 h postinjection, which is likely because of the enhanced permeability and retention effect via blood circulation^65^. The NIR fluorescence intensity in the tumor was at the same level as that in the kidneys, but much higher than in other organs at 3 h postinjection (Fig. 4c).

Based on our observations via NIR FL imaging, we examined the feasibility of using the CDs for PA imaging in vivo. Since PA and photothermal effects are typically associated with each other, another tumor model (4T1) was used for in vivo PA imaging. Mice with 4T1 tumors were intravenously injected with the CDs. PA maximum amplitude projection images and B-Scan PA images of the 4T1 tumor, which were recorded at various postinjection time points (Fig. 4d), clearly showed that the CDs accumulate uniformly in the tumor tissue with strongly contrasting PA signals via the blood circulation. The strongest PA signals were observed at 3 h postinjection, which aligns well with our NIR FL imaging observations. Similar PA images were obtained from mice with HepG2 tumors (Supplementary Fig. 7), thereby demonstrating that the CDs act as a NIR-light-triggered PA imaging agent in vivo. Based on our NIR FL imaging and PA imaging observations using the CDs, 3 h after injection of the CDs was the optimal time point for photothermal tumor ablation.

We also investigated the feasibility of using the CDs for PTT in vivo via intravenous injection. Three groups of H22-tumor-bearing mice (ten mice per group) were intravenously injected with CDs aqueous solution (1 mg mL^−1^, 200 µL). The tumor area on each mouse in the PTT treatment group was irradiated for 5 min with a 655-nm laser at a power density of 1 W cm^−2^ (3 h postinjection). The two control groups included mice that were injected CDs aqueous solution and no irradiated with a laser (CDs, C1), and the mice that were injected PBS solution and irradiated the laser (PBS + 1 W cm^−2^, C2).

**Fig. 5:**
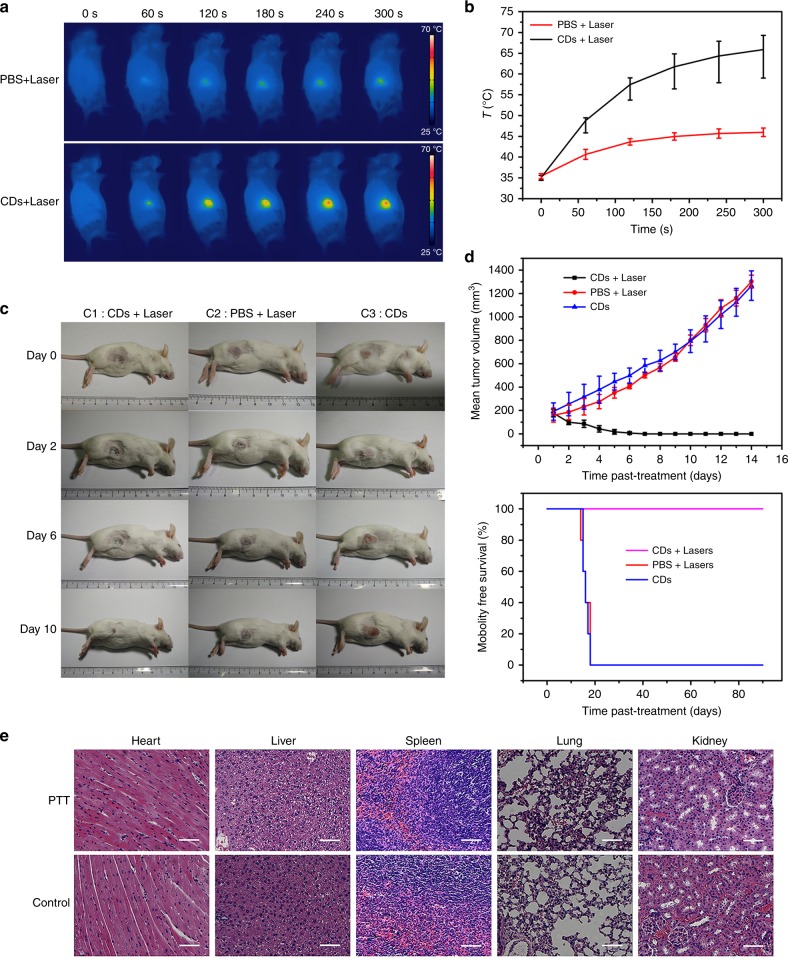
Photothermal therapy via intravenous injection based on CDs. **a** IR thermal images of mice with intravenous CDs injected at 10, 60, 120, 180, 240, and 300 s under irradiation at the tumor region by 655-nm laser at 1 W cm^−2^. **b** Temperature at mouse tumor sites as a function of the irradiation duration. **c** Photographs that document H22 tumor development on several days in live mice under various treatment conditions. **d** Tumor growth curves of H22 tumors in mice and survival rates of the groups after therapy. **e** Hematoxylin and eosin (H&E)-stained slices of heart, liver, spleen, lung, and kidney tissues of mice after PTT and control treatments. Scale bar: 50 μm

The temperature changes of the tumor area was recorded by an IR thermal mapping apparatus, which evaluate the in vivo photothermal conversion effect that was generated by the CDs. According to the IR thermographic images, the temperature at tumor area rapidly reached 55 °C in the presence of the CDs under the laser irradiation (3 min). The local tumor temperature could reached 59–71 °C after prolonged treatment (5 min), which was sufficient to irreversibly damage tumor tissues (Fig. 5a). However, under the same irradiation conditions the maximum temperature of the tumor area in the C2 group was only approximately 44 °C (Fig. 5b), thereby demonstrating that the CDs play a key role in heat generation.

The PTT efficacy of the CDs was further evaluated by monitoring the tumor growth rates. Tumor tissues in the control groups continued to grow. However, the mice in the PTT treatment group showed substantial conflagration at the tumor areas, with the tumors gradually vanishing after 8 days and the scab detaching from the skin after 10 days (Fig. 5c). Mice in the PTT treatment group survived over 3 months without tumor reoccurrence (Supplementary Fig. 8), and the mice in control groups had average life of 14–18 days (Fig. 5d). After therapy the mice body weight changes were also recorded daily, no major side effect was identified.

Meanwhile, to better understand the therapeutic effect of CDs that are used for PTT, H&E-stained sections from the major organs (kidneys, heart, liver, spleen, and lungs) of mice in all three groups were examined (Fig. 5e). No appreciable signs of organ damage or inflammatory lesions were observed in the PTT treatment group compared with the control groups (mice which were not treated with CDs) under microscopy; hence, the systemic toxicity to all these major organs was low.

## Conclusions

In summary, we developed a new type of S, N-doped CD that has intense absorption bands in the red to NIR region from citric acid, urea, and DMSO via a solvothermal method in which DMSO acts as both a solvent and an S-doping source. NIR fluorescence (centered at 720 nm) and high photothermal conversion efficiency (*η* = 59.2%) were obtained from aqueous CD solutions under 655-nm laser irradiation. The CDs, as prepared, exhibited very low cytotoxicity and were renally excreted in vivo. After intravenous injection, the CDs accumulated in the tumor tissue and exhibited strong NIR fluorescence and PA signals in vivo. More importantly, the tumors were eradicated after intravenous injection of the CDs (1 mg mL^−1^, 200 µL) followed by irradiation with a 655-nm laser (1 W cm^−2^ for 5 min). Due to these attractive properties, the CDs, as prepared for this work, can safely be used as biomedical agents for tumor FL imaging, PA imaging, and PTT. Easily synthesized CDs could relieve increasingly many cancer patients of the mental and physical suffering that is associated with cancer by providing a low-cost treatment option that has few side effects.

## Electronic supplementary material

In vivo theranostics with near-infrared-emitting carbon dots - highly efficient photothermal therapy based on passive targeting
