# Supplementary material for: In vivo theranostics with near-infrared-emitting carbon dots—highly efficient photothermal therapy based on passive targeting after intravenous administration
Source: Light Sci Appl. 2018 Nov 21;7:91. doi: 10.1038/s41377-018-0090-1 (PMC6249234; doi:10.1038/s41377-018-0090-1)
Supplement: Supplementary file 1 — In vivo theranostics with near-infrared-emitting carbon dots - highly efficient photothermal therapy based on passive targeting [file 41377_2018_90_MOESM1_ESM.docx]

Supporting Information

**In Vivo Theranostics With Near-infrared-emitting Carbon dots - Highly Efficient Photothermal Therapy Based on Passive Targeting after Intravenous Administration**

Xin Bao,^1,2^ Ye Yuan,^3^ Jingqin Chen,^4^ Bohan Zhang,^1,2^ Di Li,^1^ Ding Zhou,^1^ Pengtao Jing,^1^ Guiying Xu,^5^ Yingli Wang,^5^ Kateřina Holá,^6^ Dezhen Shen,^1^ Changfeng Wu,^3^  Liang Song,^4^ Chengbo Liu,^4^ Radek Zbořil,^6^ Songnan Qu^1^

^1^ *State Key Laboratory of Luminescence and Applications, Changchun Institute of Optics Fine Mechanics and Physics Chinese Academy of Sciences, 3888 Dong Nanhu Road, Changchun 130033, China*

*^2^School of Physical Sciences, University of Chinese Academy of Sciences, Beijing*[*100190*](http://mail.163.com/js6/read/tel:100190)*, People's Republic of China*

*^3^Department of Biomedical Engineering, Southern University of Science and Engineering, Shenzhen, Guangdong 518055, China*

*^4^Research Laboratory for Biomedical Optics and Molecular Imaging, Institute of Biomedical and Health Engineering, Shenzhen Institutes of Advanced Technology, Chinese Academy of Sciences, Shenzhen 518055, China*

*^5^Jilin Provincial Tumor Hospital, Changchun, China*

*^6^Regional Centre of Advanced Technologies and Materials, Department of Physical Chemistry, Faculty of Science, Palacky University Šlechtitelů 27, 783 71, Olomouc, Czech Republic*

Correspondence: S. Qu(email: [qusn@ciomp.ac.cn](mailto:qusn@ciomp.ac.cn)), C. Liu (email: cb.liu@siat.ac.cn) and R. Zboril(email: radek.zboril@upol.cz).


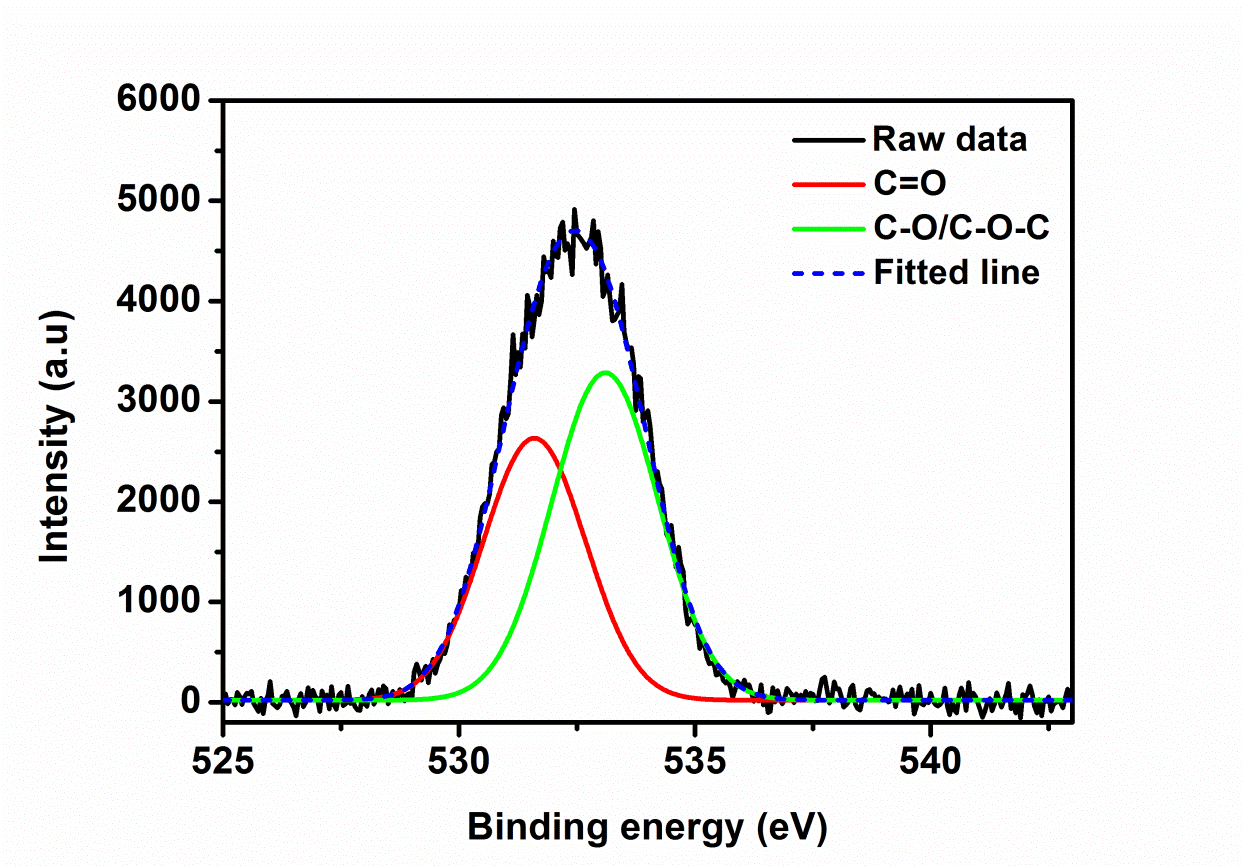


**Supplementary Figure 1** High-resolution XPS O 1s spectra from the CDs.


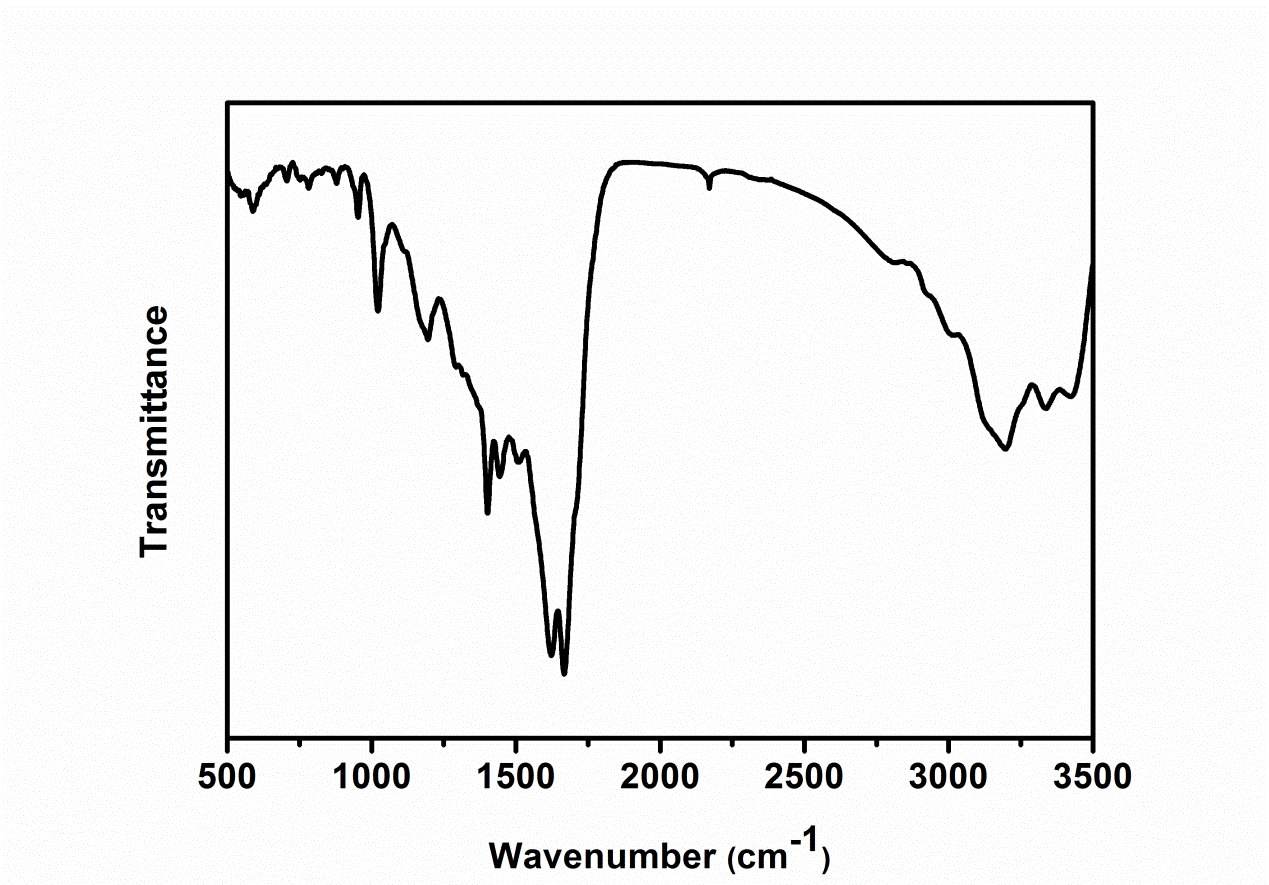


**Supplementary Figure** **2** Fourier transform infrared (FTIR) spectra of CDs.


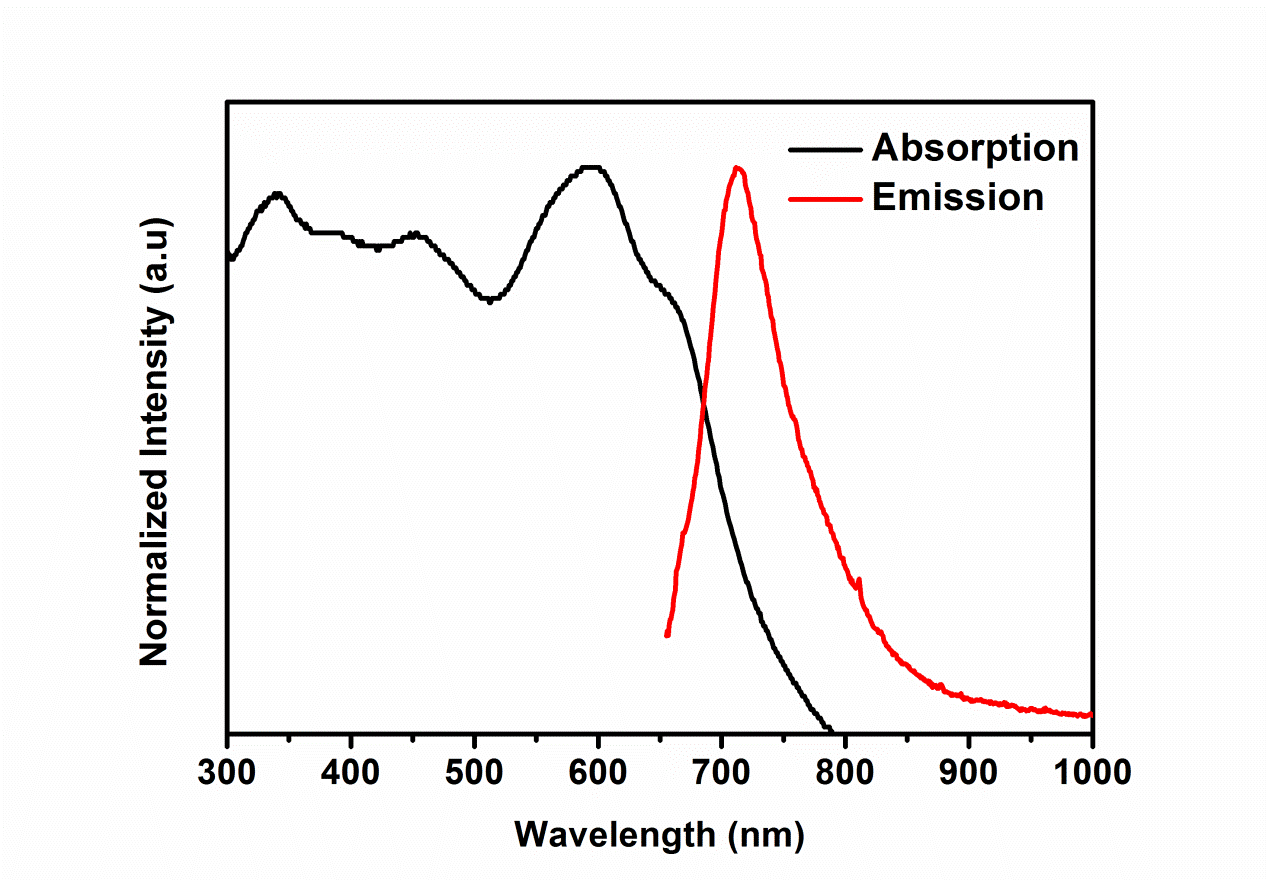


**Supplementary Figure 3** UV–vis absorption and emission spectra of a diluted CDs solution, as prepared, under 655-nm excitation.


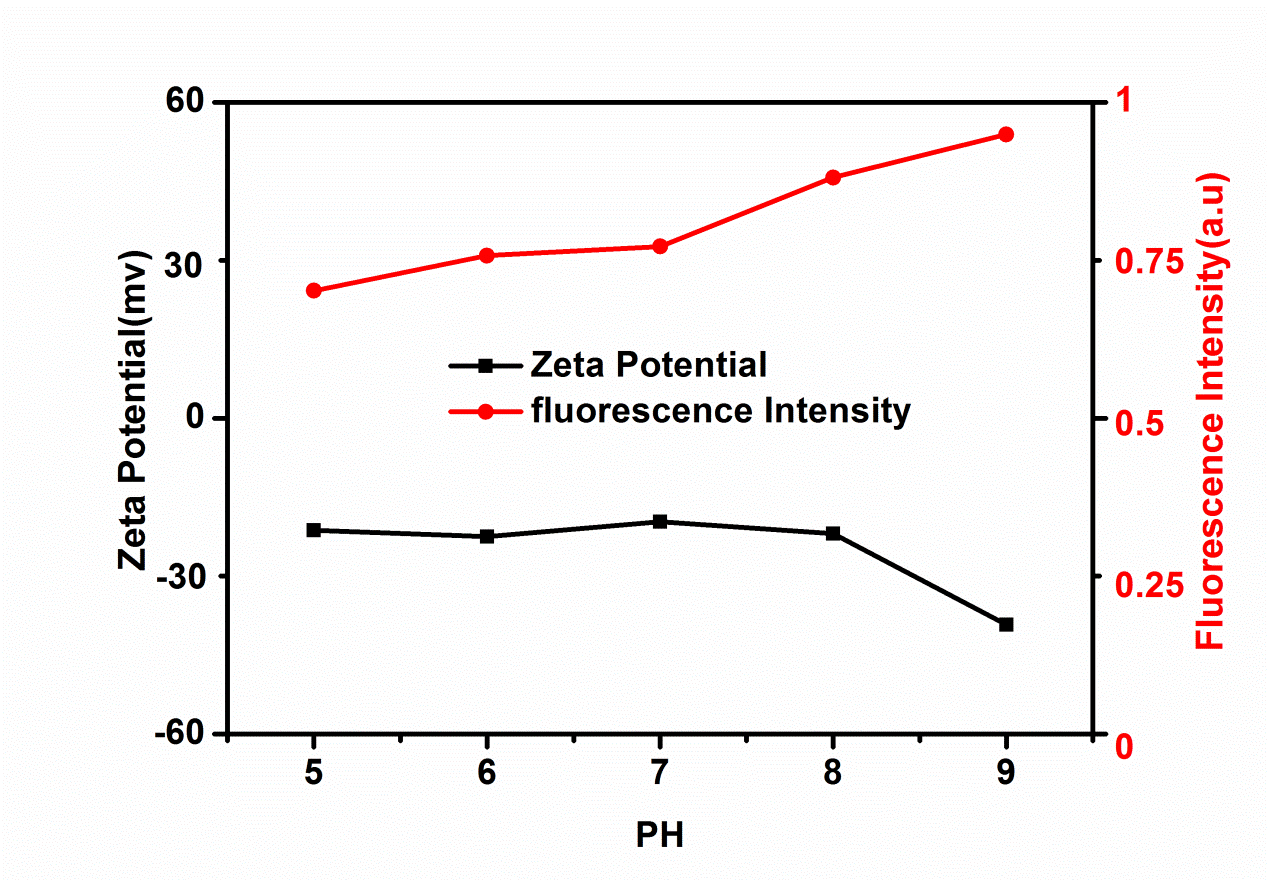


**Supplementary Figure 4** Fluorescence intensity at 720 nm and the zeta potential of the aqueous CDs solution under varying pH conditions.


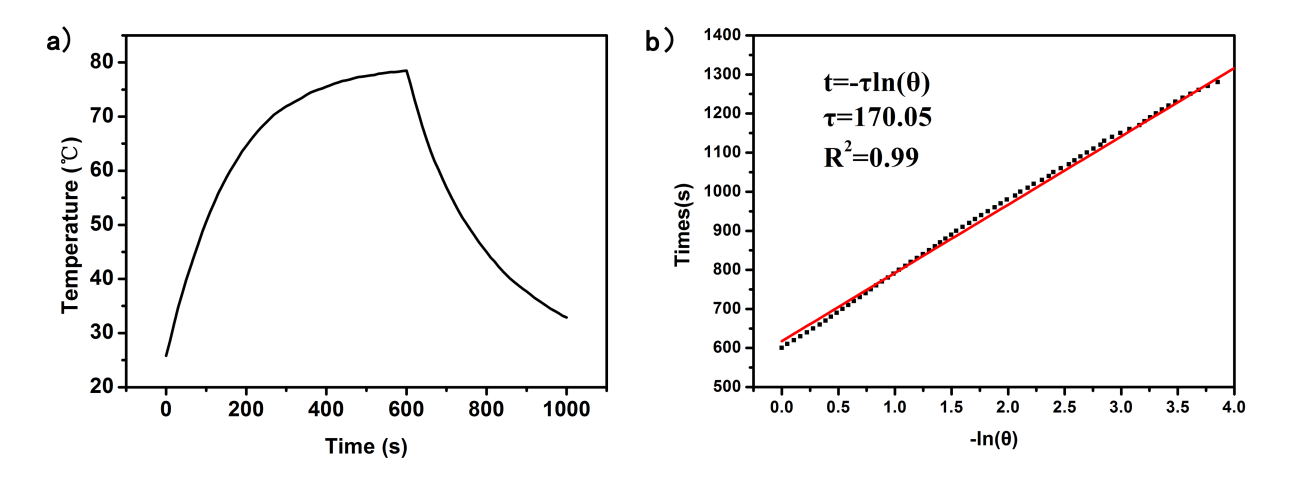


**Supplementary Figure 5** a) Temperature evolution of the aqueous CDs solution (200 μg mL^-1^) and b) linear time data versus -ln *θ* obtained from the cooling period. The temperature of the aqueous CDs solution did reach a steady state. Based on these data and previous publications, the photothermal conversion efficiency of the CDs can reach approximately 59.2%.

Photothermal conversion efficiencies were calculated as follows. From an energy balance in a system, the total energy balance can be described as:

$\sum_{i} m_{i}C_{p,i}\frac{dT}{dt}=\sum_{j} Q_{j}=Q_{I}+Q_{0}-Q_{ext}$ (Equation S1)

where the *i* terms in *m_i_*C*_p,i_* are products of the mass and heat capacity of system components, *T* is the aggregate system temperature, and *t* is time. The *j* energy term *Q_j_* includes laser-induced energy source terms *Q_I_* (from CDs) and *Q*_0_ (from solvent and container) as well as energy outputs *Q_ext_*.

*Q_I_* is the photothermal energy input from the CDs, which can be described as:

$Q_{I}=I(1-10^{-A_{\lambda}})\eta$ (Equation S2)

where *I* is incident laser power, *η* represents the efficiency of transducing incident resonant absorbance to thermal energy via plasmons, and *A_λ_* is the absorbance at the laser wavelength used (655 nm). *Q_0_* stands for the heat dissipated from light absorbed by the solvent and container, which can be measured independently using a container of aqueous samples only without CDs.

*Q_ext_*, external heat flux in the system, is nearly proportional to the linear thermal driving force, with a heat-transfer coefficient, *h*, as the proportionality constant.

$Q_{ext}=hA(T-T_{amb})$ (Equation S3)

where *h* and *A* stand for the heat transfer coefficient and surface area of the container, respectively. The product *hA* can be determined by measuring the rate of temperature decrease after removing the light source. In the absence of any laser excitation, (*Q_I_* + *Q_0_* = 0), substituting Equation (S3) with Equation (S1) gives:

$\sum_{i} m_{i}C_{p,i}\frac{dT}{dt}=-hA(T-T_{amb})$ (Equation S4)

After rearrangement and integration, the following expression for *t* is obtained:

 (Equation S5)

where *m* and *C_p_* are the mass and heat capacity of water, respectively. A dimensionless driving force temperature, θ, is introduced to get the value of *hA*, scaled using the maximum system temperature, *T_max_*, and a sample system time constant τ_s_.

$\theta=\frac{T_{amb}-T}{T_{amb}-T_{\max}}$ (Equation S6)

$\tau_{s}=\frac{mC_{p}}{hA}$ (Equation S7)

where *τ_s_* is the slope of the linear time data from the cooling period (Figure S7).

$t=-\tau_{s}\ln(\theta)$ (Equation S8)

Then the photothermal conversion efficiency (*η*) can be calculated as:

$\eta=\frac{hA(T_{\max}-T_{amb})-Q_{0}}{I(1-10^{-A\lambda})}$ (Equation S9)


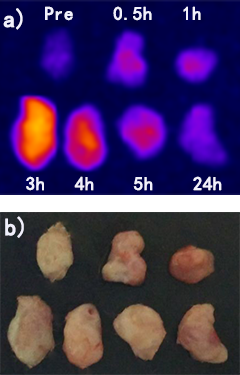


**Supplementary Figure 6** a) NIR fluorescence of H22 tumors dissected from mice at different post-injection time points. b) Photograph of excised H22 tumors under daylight.


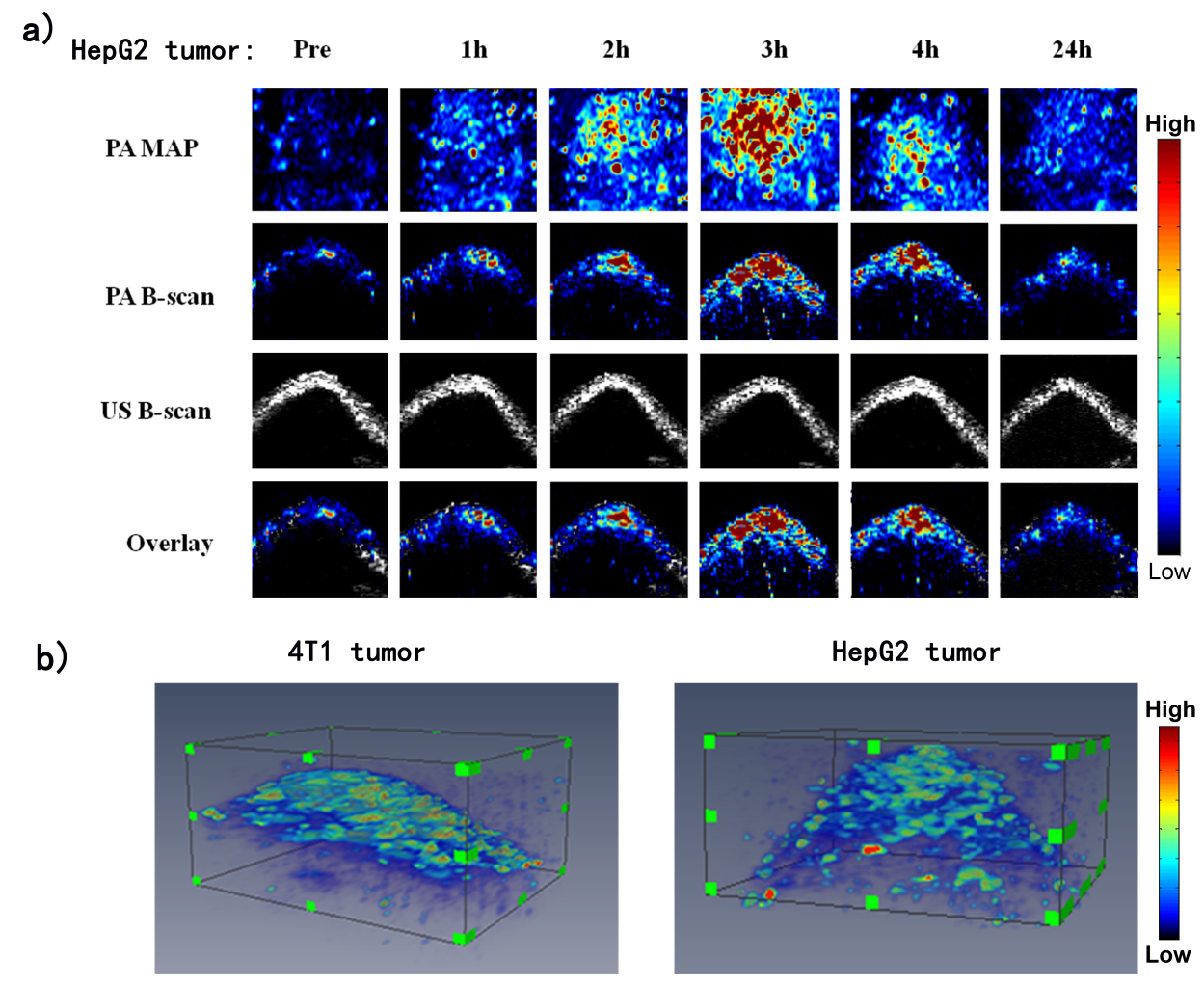


**Supplementary Figure 7** a) PA MAP images and B-scan PA images of HepG2 tumor from mice after intravenous injection with CDs at different time points. b) 3D PA images of a 4T1 tumor and a HepG2 tumor at 3 h post-injection.


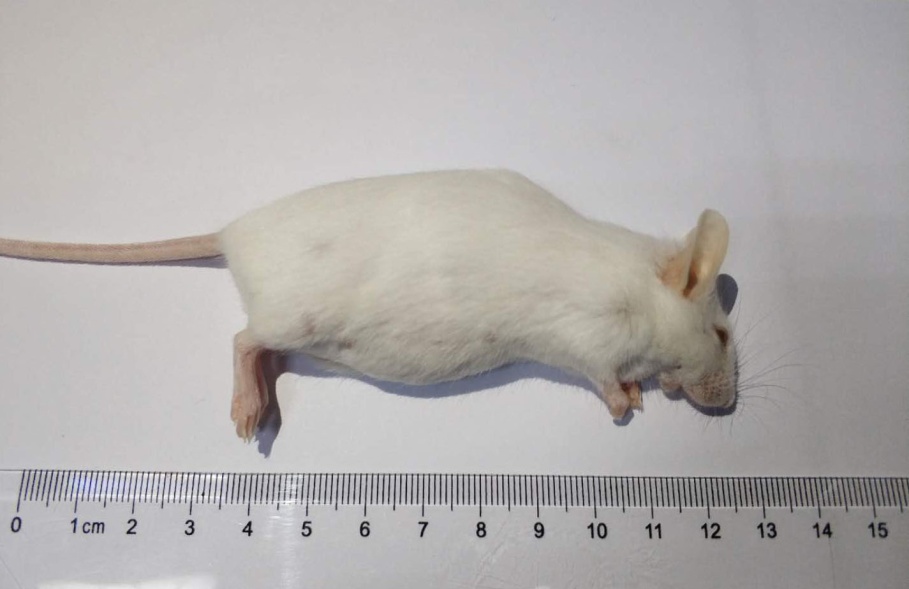


**Supplementary Figure 8** Mice belonging to the PTT treatment group were tumor-free and survived over 90 days.


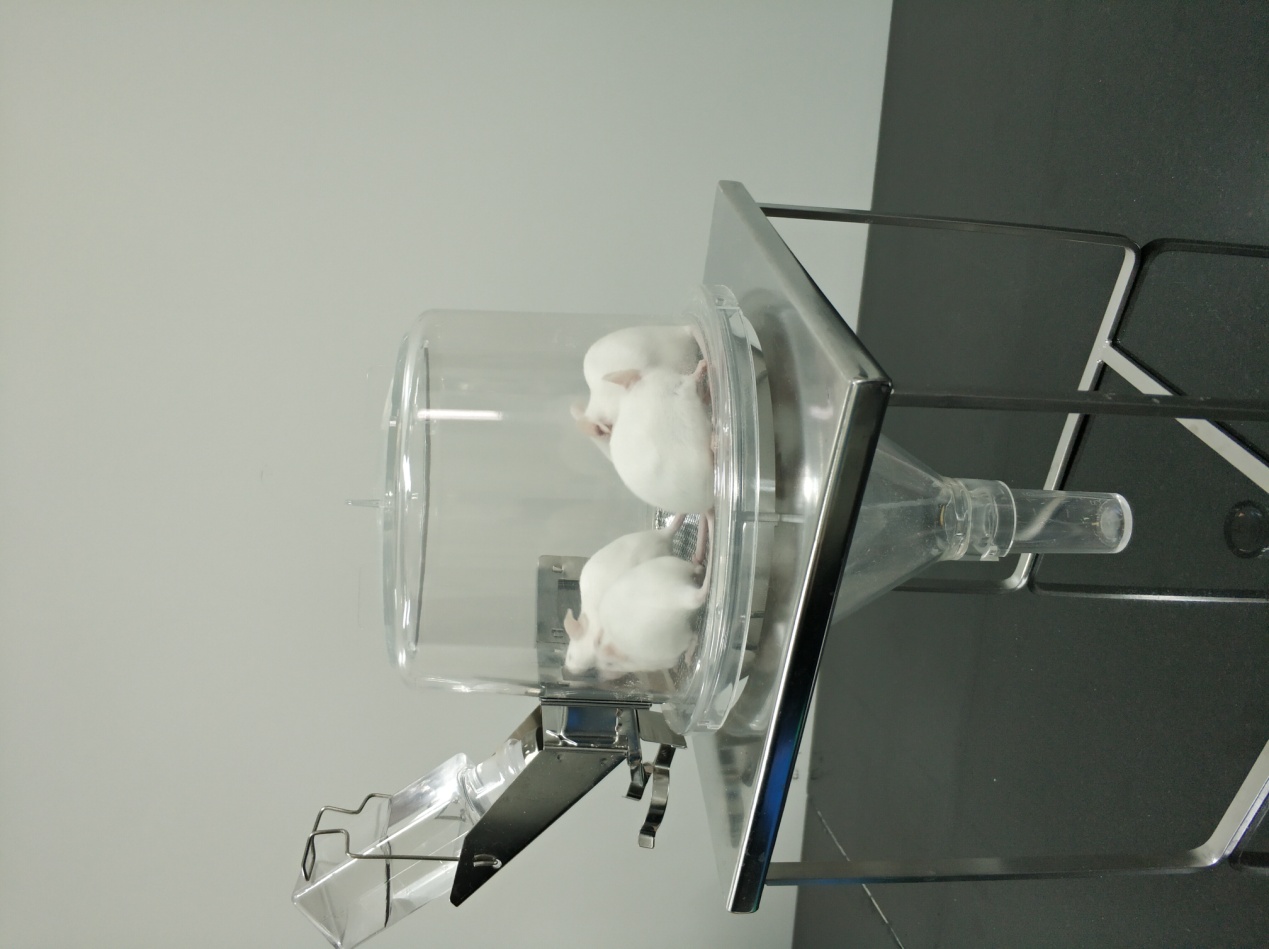


**Supplementary Figure 9** Urine collection was accomplished by using a metabolism cage, in which mice were intravenously injected CDs aqueous solution (0.2 mL, 1000 mg mL^-1^). The Urine from the mice was collected from the cup in the bottom of the metabolism cage at the corresponding time points before and after intravenous injection.


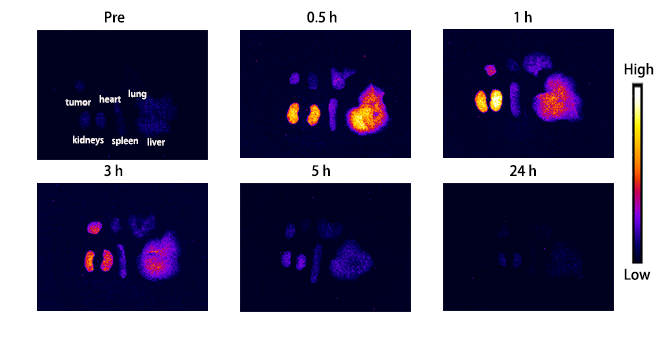


**Supplementary Figure 10** NIR fluorescence of major organs and H22 tumors dissected from mice at different post-injection time points.


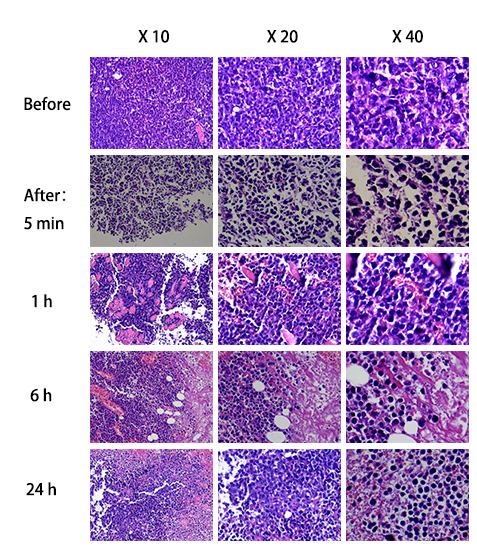


**Supplementary Figure 11** Hematoxylin and eosin (H&E)-stained slices of tumors dissected from mice with CDs intravenous injected at different time points before and after laser-treatment.
